# “It’s Still in the Test Tube and Finding out How the Experiment Ends… ”. A Qualitative Study on Health and Aging in Older Gay Males Living with HIV in England

**DOI:** 10.1177/23259582221144448

**Published:** 2023-01-03

**Authors:** Stefano Licchelli, Andrew King, Kimberley J. Smith

**Affiliations:** 1 School of Psychology, 3660 University of Surrey, Guildford, UK; 2 Department of Sociology, 3660 University of Surrey, Guildford, UK

**Keywords:** HIV, AIDS, health, ageing, homosexuality, sexuality, intersectional stigma, intersectionality, stigma, discrimination

## Abstract

This paper highlights experiences and perceptions of older gay males living with Human Immunodeficiency Virus (HIV) in relation to age, sexual orientation, HIV status and how they perceive health. Participants were gay males aged 50 and over living in England, diagnosed with HIV for longer than 2 years. In total, 19 interviews were conducted between March 2020 and March 2021. Data were analysed using thematic analysis. Three major themes were generated: 1.) Health as holistic and as a balance; 2.) The impact of HIV on people’s lives; 3.) The Intersectionality of stigma: a lifetime of discrimination. Participants highlighted the changing nature of the concept of health through their lifespan while the intersectionality of stigma in different contexts is examined considering the personal journey of living with HIV. The implications of health as a complex concept and intersectional stigma on the planning and delivering of care in this population are discussed.

## Introduction

While some research has been carried out on aging with Human Immunodeficiency Virus (HIV), there is still little scientific understanding of the experiences and needs of specific groups and the impact of intersectional characteristics on the aging experience in this population. Older people living with HIV have been considered as ‘Uncharted Territory’^1^ as they represent ‘a new aging population’ which has seen the effect on their lives of HIV becoming a chronic condition due to the new medications available.^2^ This shift from AIDS -defining illnesses to HIV as a chronic condition as seen in people living with HIV developing multiple comorbidities as they age.^3,4^ For instance, Turrini et al^4^ have highlighted how older people living with HIV have increased odds of multiple chronic conditions when compared to people living without HIV. There is currently a lack of evidence about the needs of people aging with HIV and more attention is needed to bring the subjective voices of this population^5^ and how to manage aging with HIV as a professional and as a patient.^6^ Recent statistics also suggest that the numbers of people aged 50 and older accessing HIV care has been increasing over the past few years.^7^ Thus, it is important that we conduct research into the lived experience of aging with HIV as people aged 50 and older are considered the fastest growing demographic of people living with HIV.^8^

Research is starting to show the impact that aging with HIV has on health and wellbeing. Physically, it is still debated whether HIV can be associated with accelerated or accentuated aging.^9,10,11,12,13,14^ Nevertheless, HIV has been linked to an increased risk of developing secondary co-morbidities^8,15,4^ and co-infections.^16,11,13^ Psychosocially, people aging with HIV report that they can struggle with the uncertainty of what will happen to them as they age, given their status as a new-aging population.^17^

While our understanding of aging with HIV is improving, there is a need to better understand intersectional experiences of aging with HIV. Intersectionality was originally termed by Crenshaw^18^ to define how multiple social identities can ‘intersect’ to influence how a person is treated within society, with different intersections providing unique identities, experiences and outcomes. When investigating the lived experience of this new population of gay males aging with HIV it will be important to consider how intersectional identities could shape the experiences of aging through their lifespan with a focus on gender, homosexuality, older age and HIV. In the next paragraph we will introduce examples of studies focusing on HIV from an intersectional point of view.

HIV was historically considered to be a disease which occurred primarily in gay men.^19,20^ Indeed, Haile et al^21^ explored the intersection of race, sexual orientation and socio-economic status and stigma in older black men who have sex with men living with HIV in the USA, highlighting how the achievements in the medical fields were undermined by the power of stigma in this population. According to their study, participants perceived stigma in the institutions of care as a powerful dehumanising force that would not allow them to actively influence the quality and reliability of the services offered.^21^ In another qualitative study focusing on older gay males living with HIV, it was highlighted the different kinds of stigma that can be perceived in this population that are linked with the perception of being part of minorities such as being gay, being older, having HIV and/or having mental health conditions.^22^ When considering aging, sexual orientation and HIV status, it has been highlighted a need for safe personal care which involves care delivered considering the intersection of different characteristics.^23^ Kia et al^23^ highlighted a need for gay and HIV-friendly care in contrast with unkind home-based support. From the interviews it emerged that their participants expected to encounter hostility when accessing care given the past exposure of this population to discrimination based on sexual orientation, socio economic status, HIV status and classism and this could affect their health as highlighted by recent studies which showed that internalised stigma, experienced stigma and adversarial growth negatively affected health outcomes in people aging with HIV.^24,25^

This paper explores the perceptions of older gay males living with HIV around health and wellbeing as well as their experiences as people aging with HIV. In this paper we analysed the interviews from 19 gay males living with HIV, asking them how they operationalise the concept of health while exploring how HIV has affected their lives. The interviews were analysed using thematic analysis and highlighted how the concept of health is strictly situated within the lifespan and it changes based on the change in personal circumstances in life and medical advancements as also highlighted by Persson et al.^26^ Furthermore, the analysis of the interviews highlighted how stigma and discrimination are still an important factor affecting their lives permeating different contexts such as healthcare services and the gay community.

## Materials & Method

### Study Design

A qualitative approach has been adopted for this study, using semi-structured interviews with older people living with HIV. During the interviews, the participants were asked to share their perceptions around different topics such as their idea of health, aging with HIV, discrimination and social and community determinants (Supplemental Material). The researcher conducting the interview (SL) is a PhD student with a MSc in Health Psychology and experience volunteering within charities with a focus on HIV and sexual health in both Italy and England.

### Study Participants

The inclusion criteria for taking part in the study were as follows: (1) be 50 years old or older; (2) identify as a gay male; (3) received a diagnosis of HIV at least two years before the start of the study; (4) living in the UK for at least 5 years.

Participants were recruited utilising convenience sampling via newsletters of charities and posts on social network groups (eg, Terrence Higgins trust, George House Trust, Blue Sky Trust, etc) and offered a £10 Amazon voucher for their participation in the study. A total of 19 participants were interviewed between March 2020 and March 2021, during the COVID-19 pandemic. Participants’ demographic details are summarised in Table 1.

**Table 1.** Demographic characteristics of interviewees*.*

	HIV-Positive sample (N = 19)
Mean age (years)	59
**Civil status**	**N(%)**
Single	13 (68.4%)
Married	3 (15.8%)
Civil partnership	1 (5.3%)
Widowed	1 (5.3%)
Living with my partner	1 (5.3%)
**Ethnicity**	**N(%)**
White British	12 (63.2%)
White Others	6 (31.6%)
Prefer not to say	1 (5.3%)
**Education**	**N(%)**
GCSE/O level/CSE or equivalent secondary school qualification	1 (5.3%)
‘A’ level or International Baccalaureate or other Further Education qualification	3 (15.8%)
Nursing or other medical qualification not yet mentioned	1 (5.3%)
University first degree (eg BA, BSc)	7 (36.8%)
University higher degree (eg MSc, PhD)	7 (36.8%)
**Occupation**	**N(%)**
Retired	5 (26.3%)
Part-time work	1 (5.3%)
Full-time work	6 (31.6%)
Self-employed	1 (5.3%)
Unemployed	3 (15.8%)
Prefer not to say	3 (15.8%)
**Length of time since diagnosis**	**N(%)**
At and before 1995	6 (31.6%)
Between 1996 and 2015	12 (63.2%)
At and after 2016	1 (5.3%)

### Demographic Questionnaire

A brief demographic questionnaire was designed to retrieve some basic information such as age, sexual orientation, relationship status, ethnicity, level of education, occupation status and year of diagnosis.

### Semi-Structured Interview

The semi-structured interview schedule investigates the perceptions and personal beliefs of participants around some areas such as personal definition of health, aging with HIV, discrimination, temporal perception of living with HIV, social and community determinants. A full list of questions and prompts is given in the Supplemental Material.

### Ethical Approval

This study has been conducted after receiving favourable ethical approval by the University of Surrey (ref: FHMS 19-20 037 EGA). All participants provided consent to take part in the study agreeing to an online version of the informed consent form. To protect participant’s identity, personal information was stored on password protected folders within the University. Participants received a debriefing at the end of the interview to minimise the possible impact of the research questions on their wellbeing.

### Interview Procedure

Participants interested in taking part in the study contacted the researcher via e-mail and the researcher sent them a copy of the Participants Information Sheet and consent form along with a link to a Qualtrics survey. The survey contains a digitised version of the Participants Information Sheet, consent form, the demographic survey and a table where participants were able to state their availability during the week. In order to take part in the study, all participants signed the digitised version of the consent form.

After that, participants were contacted to arrange a date for the interview to take place. Interviews were conducted on Skype, audio recorded and transcribed verbatim. After transcribing, the transcript was anonymised and the audio file deleted. The mean length of interviews was 36 min (range 11 min to 2 h and 14 min). During the interview or immediately after, the researcher would fill in a self-reflective form inspired by the cognitive model presented by Padesky and Mooney^27^ if strong emotions or thoughts emerged during the interview to improve reflectivity. At the end of the interview, a debriefing was conducted by the researcher.

### Analysis

Anonymised transcripts were analysed using thematic analysis.^28^ The analysis followed an inductive process, where the transcripts guided the generation of themes. For the data analysis, we used QSR International’s NVivo 11.4.1.1064 software to highlight quotes and generate themes. The first author familiarised himself with the transcripts, reading them multiple times and highlighting important quotations in relation to the research question. Subsequently, the researcher generated initial codes and indicative themes from the transcript. A review of the codes and possible themes was conducted during a meeting between the authors. After that, a first thematic map was drafted and discussed between the authors. The draft of the thematic map was then sent to participants for feedback. The final version of the thematic map was created after review of the feedback between the authors.

In a second step to the analysis, a draft of the thematic map was sent to participants who consented to be contacted for feedback. A total of 19 participants read through the map, and 2 provided feedback. The feedback provided by participants indicated that the concept of spirituality in health needed to be expanded and that the sub-theme related with the expectation towards the healthcare needed to be reframed. This feedback was reviewed by all study authors and integrated into the final version of the thematic map.

## Results

### Overview

From the analysis of the interviews, it was possible to highlight four main themes on how participants make sense of what health means in relation to their life experience living and aging with HIV. A common element emerged through the four main themes of aging with HIV as a journey. Participants reported their experiences of living with HIV throughout their lifespan, presenting their personal journey through life living with HIV.

Three major themes were generated: the first theme, health as a holistic balance, where participants highlighted what health means for them, how the idea of health has changed through their lives and how good or bad health impact their ability to do what they value as important. The second theme, the impact of HIV on people’s lives, considers the impact of HIV on people’s lives, exploring what living with HIV means from a social point of view but also how medical advancements affected HIV considering both the change from a deadly to a chronic disease and socially with the Pre-Exposure Prophylaxis (PrEP) and Untransmittable = Undetectable (U = U) campaign. The last theme was generated considering the experiences around and perception of stigma in different contexts and the effects of misinformation in relation to HIV. For an overview of major themes and subthemes see Table 2.

**Table 2.** Summary of major Themes and Related Sub-themes.

Main Theme	Sub-theme
Health as holistic and as a balance	Health as an idea that changes over time
	Health as not just an absence of illness
	Health as a feeling within the body
	Good health as a resource to do what is important
The impact of HIV on people’s lives	HIV throughout the lifespan
	The medical journey with HIV
The Intersectionality of stigma: a lifetime of discrimination	Myths and (mis)information

### Health as Holistic and as a Balance

During the interviews, participants were asked to define health and what good and bad health means to them. A range of different answers were provided and interviewees highlighted how health is a complex concept, not easily definable. However, health was seen as the interlinking of both physical and mental health and as a balance that allows participants to do what they choose. One participant has highlighted how mental and physical health are interlinked and a change in one may cause a change in the other.

“*so for me health is very holistic in the sense that includes what I am able to do…and there is no restrictions on that…my mental health improves as well as my physical health so the two are quite interlinked for me…” [Participant 10, 57 yo]*

As reported by Participant 10, mental health is an important element and this has also been highlighted in the literature.^23^ This interlinking balance of physical and mental health is seen as being unique and tailored to the individual highlighting how different people might have different and personalised views around what can be considered health and being healthy and how they can experience health:

“*I mean because I mean I am one of those who think that ok you can be healthy and you can still have a cream cake every now and then if you were having that everyday then it is not going to be healthy. It is a balance. Anything in moderation.”[Participant 1, 58 yo]*

Health is operationalised as a holistic balance while at the same time maintaining the possibility to be tailored around personal and individual differences. In addition, the idea of health allows exceptions and it is not rigidly confined to set rules aimed at prohibiting what can be done. Health does admit pleasure and does allow “*a cream cake every now and then”* [Participant 1, 58 yo] assuming that the person is able to keep a balance and that a balance is maintained.

Nevertheless, other definitions of health emerged from the interviews of participants. Health might involve the consideration of how HIV and other illnesses are seen as well as the dominant definitions of health that are available and socially accepted. For example, one participant reported

“*I am not sure what kind of definition you want…you can have a medical definition of health which is based on your blood count and your blood pressure and all those things….that*’*s a very scientific and straightforward but health is about being able to interact and to be able to pursue what you want to do and to operate independently.” [Participant 4, 69 yo]*

Rather than excluding completely or discarding the “*medical definition of health* ” *[Participant 4, 69 yo]*, Participant 4 has acknowledged that health is defined in multiple ways but the way participants in this study perceived health is mostly related with a feeling of holistic balance enabling them to do what they value.

This theme highlights how health is not fixed or generalisable but is more personal, suggesting that it would be important to consider subjective views for example when planning people’s care.

### Health as an Idea That Changes Over Time

Participants highlighted how the idea of health has changed throughout their lives, especially comparing how they perceive health now with when they were young. Some participants reported how:

“*when you are young you are immortal aren*’*t you? You don*’*t think you are ever going to die and it doesn*’*t even enter into your mind does it? And the idea of living to be a forty is just a ridiculous idea, isn*’*t it? So you really take much stock in your health and you take more risks…so yes, the approach to health changes as you mature, as you start to feel your mortality…..” [Participant 8, 54 yo]*

*“when I was younger…[…] I could easily go out after work every night of the week and still get up for work the next morning and it had no impact or very little impact…nowadays, no way…if I go out for a night out it takes me a week to recover [laugh]…so yeah…my concept of health has changed significantly…” [Participant 10, 57 yo]*

Participants 8 and 10 highlighted how they perceive more their mortality compared with a younger age when a feeling of invincibility was perceived. The participants have reported some anecdotes of their lives suggesting that health in younger age was considered as more related with being fit, having more energy and vitality while now health is seen more as a balance and the approach to health had changed because of a change in the position or circumstances of the person. Their idea of health has changed over time suggesting that health might not be a fixed and stable construct but more flexible and variable through the lifespan of each individual resonating more with that idea of a concept of health tailored around the individual and their personal history. Thus, the interventions and policies in place to promote health for older gay males aging with HIV now might not be the considered appropriate in the future and the idea of health and what people define as healthy should be monitored over time. This links to the next sub-theme of health as not just absence of illness.

### Health as not Just an Absence of Illness

This change in how health is perceived throughout the lifespan also applies to how illnesses are seen. Living with HIV for some people does not necessarily mean not being healthy. For example, a participant reported that

“*I realise that I can be healthy and still be HIV positive…you know…so I am living with HIV but I am still healthy…. ” [Participant 9, 58 yo]*

The idea of health is influenced by the aging process and changes to suit the reality of living with a chronic condition highlighting how having an illness does not preclude health. There is a realisation that health does not exclude illnesses and having a chronic illness does not exclude the possibility of being healthy, resonating with the idea that “*the approach to health changes as you mature*” [Participant 8, 54 yo].

On the other hand, some participants also highlighted how their idea of health is linked with the concept of illness and how these two are interlinked. Illnesses can interrupt that personal experience of “feeling healthy” as described by Bury^29^ when discussing chronic illness as biographical disruption, highlighting how chronic conditions can disrupt the “normal” or established structures of everyday life.

“*Resignation comes into being resigned to this level as opposed to this level [of health]….in a way that comes with a lot of stuff…I have spent a lot of my life looking ahead to try and get to here rather than just accepting, this is my reality and dealing with it…if that makes sense….” [Participant 18, 52 yo]*

Participant 18 has highlighted this point, reporting how during his experience HIV can create different levels of health where the highest steps can never be reached and the person needs to accept that they will not be able to reach the highest levels of health. Indeed, there is a resignation to a certain and “normal” level of health, that cannot be reached once you have a chronic condition such as HIV, to another sub-optimal level of health. This point is particularly important as it highlights the negotiable nature of health as also emerged in Persson et al.^26^ The authors highlighted how the concept of health is never given once and for all but it is constructed and re-defined based on medical advancements and changes in the personal lives of people.

Health is more than the absence of illness and a connection was highlighted by some participants in relation to spirituality and a general sense of fulfilment.

“*accordingly to the stoic philosophy health is something that you would prefer to have but it is an indifferent so in technical terms it means that…it is indifferent compared to “virtue”, virtue is a technical term, it is just means that as long as, in a nutshell, you are a nice person, health is irrelevant and money is irrelevant […]so for me this was very empowering because I thought ‘well, my health status I obviously can*’*t change but can I make myself a better person? Of course I can’” [Participant 11, 52 yo]*

Health can also be found within the concepts of spirituality or personal philosophies which can support the individual during their journey and empower them providing an alternative framework used to give sense to the world and what happens in everyday life as reported by Participant 11.

In this sub-theme participants have highlighted how illnesses are not necessarily to be considered as antinomies of health and that the personal perspectives around health shapes their attitude towards what it means to be unwell.

### Health as a Feeling Within the Body

Health is also presented by participants as a feeling perceived within the body. This feeling is used by people to inform how there are feeling in terms of good or bad health as reported by these participants

“*so when I find I have a lot pain and I am concentrating on that, that is not good health…good health means that you almost ignore what it is happening to your body and instead you are enjoying to the sensory perceptions…[…]” [Participant 4, 69 yo]*

*“Q: What are the things that make you said that you have a good or bad health?*

*A: I think it is just….I think it is just ahem…mmm…nice question…I think it is a matter of…it is very difficult to define….I think it is a matter of feeling good, and some morning you wake up and you leap up out of bed and the world looks wonderful particularly when it is sunny you know what I mean […]” [Participant 5, 73 yo]*

Participant 4 highlighted how he identifies the focus on pain as a sign of ill health, while being able to focus on the world and the surrounding environment is seen as a sign of good health. Interestingly, Participant 4 mentioned a difference in whether they are focusing on enjoyable sensory perceptions instead of focusing on negative physical perceptions and how this would affect their behaviour and relationship with the surrounding physical and social environment. At the same time, Participant 5 highlights good health as a feeling that would inform the person of how they are in terms of both physical and psychological health. The concept of health seems to emerge from how health is experienced within the body first and operationalised using the feeling perceived through the body and the senses. This has also been highlighted by Persson et al,^26^ where they reported how bodily sensations help in negotiating and shaping the concept of health.

### Good Health as a Resource to do What is Important

Considering health as a feeling from within the body that informs people around their health status, participants also defined good health as a resource that they can use to do what is valued as important by them.

“*good health is being in a position where you can do what you need to do and what you want to do and being able to go to work and enjoying yourself and have friends and poor health is the opposite of that, it is having problems at employment, happiness and housing…” [Participant 7, 52 yo]*

*“My conception of health being able to do the things I want to do…ehm…”[Participant 10, 57 yo]*

Good health is perceived and described by Participants 7 and 10 as a resource that would allow them to do what they need and want to do independently. This enables participants to achieve the things they want to do without limitations, putting them in the position of being able to independently do what they want to do in their daily lives. When considering good health as a resource, it is possible to notice how good health is tailored around the individual needs and what they want and can achieve. This state of being in good or bad health affects people in their daily activities, linking health as a feeling and health as a resource. From the analysis of this sub-theme it is possible to see how the concept of health emerging from the interviews goes beyond the absence of illnesses, incorporating personal motivations and choices.

## The Impact of HIV on People’s lives

HIV is acknowledged to be part of people’s lives to differing extents. Participants have presented their experiences in relation to living with HIV and these experiences can be linked to variables such as how long people have been living with HIV, their age and their exposure to old fashioned campaigns tackling the AIDS pandemic during the 80’s and 90’s.

“*I am [in my 50s] now, I was [in my 20s] when I was diagnosed with HIV, it*’*s all my life really, it*’*s almost impossible to separate the two…. ” [Participant 3,56 yo]*

*“ultimately I can*’*t remember a time where I wasn*’*t positive…you know, my entire adult life I have been HIV positive, most of it….so again, there has never been a time where I haven*’*t been taking tablets, there has never haven*’*t never been a time where I haven*’*t had side effects, there has never been a time where…[…] it is always there in the background, sometimes in the forefront, but it is always there, always present…” [Participant 18, 52 yo]*

Participants 3 and 18 have highlighted the common idea that HIV has been part of their lives to such an extent that it is impossible to recall a time when the person was living without HIV. Indeed, there is a need to differentiate the experiences of people who have been aging with HIV and people who have acquired HIV at older age. For the participants who have aged with HIV, their lifelong journey is a journey with HIV and that journey is strictly linked with the advancements in the medical field for example with the discovery of new classes of antiretrovirals as well as with the management of possible side effects due to the medications. For the first time we are seeing people living with HIV aging with HIV and some participants strongly perceived this sense of uncertainty as the first generation to face the aging process with HIV.

“*for somebody like myself there isn*’*t really anybody about who has lived longer with the virus so I am part of a generation who are finding out precisely what the correlation between health, HIV, age, sexuality it*’*s still in the test tube and finding out how the experiment ends… ” [Participant 3, 56 yo]*

A feeling of uncertainty that is given by the perception that this first generation of older people aged with HIV is the first to face the effect of living longer with the virus and this is seen as impacting in multiple ways. In the next paragraphs I will present the subthemes linked with the impact of HIV on people’s lives emerging from participants’ interviews.

### HIV Throughout the Lifespan

The journey with HIV throughout the lifespan is strictly linked with the changes and advancements in the medical field that brought a change in the outcome of living with HIV from deadly to a chronic condition and the effects of this change on people’s lives. Indeed, participants have reported the following:

“*so the perception that I started with was ‘we are all going to die’ and then I was in a marriage with a guy for 13 years and […] when that ended I had to re-educated myself as what it meant to be positive and to understand this U* *=* *U thing…” [Participant 13, 60 yo]*

*“I mean they probably don*’*t realise that people are still dying of HIV in other parts of the World especially in Africa…we are lucky in the UK and Europe that most of us are very healthy because we are on the correct medications and things like that” [Participant 9, 58 yo]*

The perception participants had when they were first diagnosed with HIV was that of HIV as a deadly condition as highlighted by Participant 13. However, the outcome of the condition has changed and people needed to re-educate themselves when thinking about HIV and challenge the idea of HIV as a deadly condition. For example, the U = U campaign is spreading the message that people living with HIV with undetectable viral load are not able to transmit the virus.^30^ This is possible due to the medications available and to the healthcare systems in Western Countries as highlighted by Participant 9. The change in outcome of HIV is possible due to the investment in public and free healthcare where medications are freely available for the users. Indeed, it is possible to highlight how different people living with HIV might experience different trajectories through their lifespan considering the availability or medications as well as the timely diagnosis of their condition. Furthermore, participants highlighted once more how the changes in their lives affect their health and how their idea of health needs to adapt when advancements in the medical field bring changes in the outcome of lives of older gay males living with HIV. Hence, reinforcing that idea of circularity highlighted by Persson et al^26^ in their study.

Indeed, this point is going to be explored more in detail in the next paragraphs where we will present how the medical journey affects the personal journey of participants.

### The Medical Journey with HIV

This subtheme focuses more on the perception of the medical side of the journey of living with HIV. We have seen in the previous subthemes how advancements in the medical field have had an effect on the lives of participants. However, participants highlighted different views and how there is uncertainty around the link between HIV and aging and it is not clear if HIV contributes to the process of aging and if so, to what extent as also highlighted by Rosenfeld et al,^6^. Participants have reported:

“*We have accumulated bad habits as we get older, we might not have looked after ourselves, we might have abused our body in all kind of ways…people are very fearful about long term effects of medications and there is no evidence…and now people have been on medications for 30 years and there is no evidence to show that long term medication is having a negative effect in a significant enough…impact to really change things…lifestyle factors need to be taken into consideration as well…” [Participant 5, 73 yo]*

*“I suppose…anything that happens to you, you are wondering ‘is this just normal aging or is this HIV related problems?’ so then it is harder to see how much you want to push to resolve it or you have just to accept that this is part of a natural process…” [Participant 13, 60 yo]*

It is possible to see how there are different views when considering the interlinking of aging and HIV as also highlighted in the literature.^31^ While some people focus more on bad habits and the lack of evidence around long-term effects of medication, other interviewees remain more unsure on how much their issues are happening because of their age or because of HIV. For example, Participant 5 reports how the scientific evidence around this point shows how long-term use of medications do not have a significant impact on the journey of people living with HIV. While Participant 13 feels that it is not clear what kind of link there is, if any link exists at all, around the effect of HIV on aging. Indeed, there are opposite views and research is still working trying to explore and understand this new relation between HIV and aging. Furthermore, the impact of bad habits on health needs to be taken into account when considering long term effects of HIV on aging.

Other physical health issues that are part of this medical journey were highlighted by participants such as conditions other than HIV and perception of side effects of the HIV medications.

“*I worry that one day my liver may pack in and my kidneys may pack in because of the long term effects of the combination therapy that is made of very strong medication after all…” [Participant 8, 54 yo]*

*“Well, it is related to age as well as HIV…Probably more towards age because I now have various issues…[…] I used to drive and I can*’*t drive now, they took my driving licence away…I have no peripheral vision…those kind of things impact on my health and they make more of a challenge you see…” [Participant 4, 69 yo]*

Participants presented a more systemic picture of their condition with the perceived interlinking effects of medications on the body and other health conditions. Participant 8 highlighted a general sense of concern around the long-time effect of medications on the body and how to deal with this. For instance, HIV medications are perceived as strong medications that in the long term can lead to comorbidities and other physical problems which, in turn, can lead to a decline in functional abilities. Physical problems interrupt the “normal” flow of people’s lives, impacting on their ability to do things which, as we have seen analysing the previous theme, is at the core of the concept of health as defined by participants and this might also be linked with what Bury^29^ has highlighted when discussing chronic illness as biographical disruption which involves the mobilisation of resources to face the change in situation of the individual due to the chronic condition. Furthermore, participants reported health problems other than HIV which may or may not be linked to HIV, complicating the picture in terms of actions to be taken to tackle these other medical issues.

This interlinking of HIV, aging and effects of the medications is something that is still under exploration and as shown by the interviewees it is something important for participants. Based on the foregoing analysis, it is important to provide clear answers around this topic as well as a clear understanding of what the link is between HIV and aging while considering and promoting healthy habits and positive aging, to make sure that older gay males living with HIV are in the position to live an independent and healthy life.

## The Intersectionality of Stigma: a Lifetime of Discrimination

The multidimensionality and complexity of the concept of stigma in this population has already been established in the literature^21,31^. With this theme I introduce the intersectionality of stigma considering sexuality, aging and HIV status in different contexts. The extracts below are exemplificative of discrimination perceived by some of the interviewees:

“*well actually dirty, people ask me if I am ‘clean’, you know that for me is quite…I don*’*t understand how people associate being HIV-negative with being clean…” [Participant 16, 54 yo]*

*“I think it is little things like people ‘oh, should you be climbing ladders at your age?’ or a nurse saying…I was taking the rubbish out for my mother, before she died, I slipped on the ice and I cracked a rib and this stupid nurse goes like “oh, once you’ve fallen you will fall again” just because you are 60 something or 70…[…]” [Participant 5, 73 yo]*

*“it not discrimination but, when I see young people and they have a different attitude towards older people…it is not discrimination but I am a bit pissed off…you know, when they distribute flyers in a night club and they give to everyone around except me and they might think ‘this guy is not part of the age rang’ ” [Participant 19, 65 yo]*

From the analysis of the interviews it is possible to see how discrimination can take multiple forms and can be perceived as targeting multiple levels of people’s identity in contrast with that highlighted by Emlet^32^ where the focus of stigma was on aging and HIV status and their intersection. Participant 16 has highlighted how discrimination based on his HIV status is still alive, especially when using web apps for dating, where it is still possible to see others referring to a person living with HIV as *“dirty”* which, as highlighted by Spieldenner^33^ and as experienced by the participants, is value-laden. It is also possible to see discrimination based on ageism as described by Participants 5 and 19 and how older people are considered as at risk simply because of their age or are excluded from taking part in certain events because they are not seen as or considered as appropriate for inclusion due to their age. The perception of older age seems to be based on assumptions of what older people are allowed to do and which events they are allowed to participate in, with a possible impact on what events and activities older gay males living with HIV are supposed to attend or join in.

Furthermore, after highlighting different forms of discrimination, participants have reported how these are particularly present in some settings such as the gay scene, healthcare settings and more rural areas. In the next paragraphs I will briefly present how participants have reported discriminations in these different settings.

“*I think there is still a huge amount of discrimination around, in certain sections of society and actually in the gay community there is probably still discrimination about it coupled with the inherent discrimination against aging, especially in the gay community….” [Participant 7, 52 yo]*

*“one time this nurse said to me, only two years ago ‘oh, you’re HIV positive….don*’*t you look well?’ as if I have to look like I am dead because I am HIV positive…the presumption again is a bit like old age….the presumptions, you know, you are old, you live with HIV, you are a miserable old sod ….” [Participant 5, 73 yo]*

Participant 7 highlighted a generalised feeling of discrimination perceived within the gay scene where it is possible to find stigma against people living with HIV and older people. Johnson Shen et al^34^ have speculated that this is due to cultural norms and a more positive view of youth within the gay community. In certain settings, HIV is seen as an element of social and moral judgement, where “clean” people are better than “dirty” as highlighted by Spieldenner^33^ or where HIV can be used to judge another person and their level of “promiscuity”.

It is also possible to see something similar happening in healthcare settings, as reported by Participant 5. Some healthcare professionals might still make judgements based on old preconceptions around HIV and on the assumption that it is possible to judge how an older person living with HIV should look like and the physical characteristics that would identify them or label them as “*miserable old sod*” [Participant 5, 73 yo].

It is important to tackle these different forms of discrimination that affects the experience of the journey of living with HIV and the psychological wellbeing which, as we have seen above, it is an important element of health. We have highlighted how stigma is not just multidimensional but intersectional, with the potential of affecting multiple identities of the individual in different contexts. In the next paragraph, we will introduce the effects of myths and misinformation in relation to aging with HIV.

### Myths and (mis)Information

Overall, considering the impact of HIV on people’s lives, it is not possible to ignore the power of information, misinformation and myths around HIV and around people living with HIV.

“*occasionally there is still stuff popping up in the press around it…although the press is polite about it, there is still a lot of ignorance around it….although you have got things like PrEP coming out the market though even though the press fairly recently said if you take PrEP you will be going out shagging yourself senseless and catching something else…” [Participant 7, 52 yo]*

*“all the last 30 years of negative media coverage, let*’*s put it like that… it is…when you think of some of the headlines, some of the things that have been said and some of the things that are still being said, it is terrible…” [Participant 5, 73 yo]*

It has been highlighted how the media coverage of HIV has affected how the virus was seen socially and some of the headlines have been described as “*terrible*” by Participant 5. Even today, Participant 7 has highlighted how when reporting some advancement in the medical field such as PrEP, some tabloids or journals lean towards moral judgemental headlines which contributed to create once again a specific social view around HIV. One participant has reported that *“misinformation and urban myths have lot to answer for and it is a huge burden”* [Participant 5, 73 yo] on people’s lives. Myths and misinformation are significant fuel for ignorance and stigma and they can mislead the reality of scientific information while feeding into old knowledge or preconceptions. For instance, Hedge et al^35^ have highlighted how media are perceived to sustain old knowledge when considering HIV while the words used by journalists were considered to vehicle a negative image of people living with HIV. This study highlights how participants perceive this to have an effect directly on the lives of older gay males living with HIV, affecting how they are perceived on a social level.

This subtheme has highlighted how myths and misinformation have a serious and strong impact on the lives of people living with HIV when considering how HIV is seen socially and the care connected with living with HIV.

## Discussion

This paper identifies how older gay males living with HIV make sense of health and wellbeing in relation to their journey living with HIV. The themes that emerged from this study reinforced the literature around aging with HIV and present new insights. Overall, this thematic analysis has highlighted how our interviewees operationalise health as an holistic balance between the physical and the psychological side while also considering their personal journey living with HIV. Furthermore, we highlighted the intersectionality of stigma considering age, sexuality and HIV status in different settings.

To our knowledge, this is one of the few studies inviting older gay males living with HIV to define what they mean with health and how they operationalise health. For instance, before this paper, Persson et al^26^ have published a critique exploring how people living with HIV make sense of the concept of health, highlighting how *“health and illness are always contested rather than coherent, always emergent and changeable rather than determined.”* (p. 411). From our analysis emerged how health is seen by participants as holistic and as a dynamic balance between physical and mental health that changes through the lifespan, hence highlighting the importance of mental health and psycho-social factors in this population in line with the literature.^36,37,38,23^ Hence, these findings highlight how health cannot be merely considered as one or more medications that need to be taken daily but it is something socially constructed and emerging from the experiences of people living with their condition in their context. It is not possible to consider health without looking at the individual and their personal and unique journey while living and aging with their HIV.

Furthermore, we have seen how the concept of health has changed over time and this might be related with the change of HIV from deadly disease to chronic condition. It is possible to see this even more when considering the U = U campaign, where medical knowledge is translated into a social message that is perceived as liberating. Further research should be undertaken to investigate the effect on U = U message on people living with HIV and it might be interesting to consider the use of the U = U campaign in tackling stigma within the healthcare system and in the general population. Policies aimed to protect and improve health in this population need to take into account what health means for people in the context where they live to plan viable and effective intervention to support older gay males living with HIV in their journey.

Overall, this article strengthened the idea that an involvement of healthcare professionals in the views held by the participants around what they mean with health and what they value as important in their lives is needed and sought by participants. Bristowe et al^39^ have highlighted in their study the biomedical nature of the HIV consultation, highlighting how other problems are seen as secondary and not discussed during routine consultations. However, considering the analysis conducted in this paper, it might be possible to say that signs and symptoms need to be re-connected to the journey living with HIV of the individual and contextualised in light of what the person values as important in the context where they live with the support of a multidisciplinary team of healthcare professionals. In order to achieve this, it might be possible to integrate a more psychological workforce in sexual health clinics and involve more psychological professionals in the care of people living and aging with HIV as well as allowing clinicians to extend the time dedicated for routine check-ups of people living with HIV to investigating psychological and physical wellbeing of patients.

Furthermore, it has been highlighted how participants hold different views when considering the interaction of HIV and aging. Participants have highlighted how the link between HIV and aging is not clear and how this is affecting their aging process or vice versa as also find in the literature.^31,40^ Indeed, participants reported the feeling of how the intersection between HIV, sexuality and aging is still in development and more studies are needed to explore the intersection of those elements from a medical and psychosocial point of view. It is important that the interaction of HIV and aging is studied and analysed from multiple perspectives to answer clearly to the doubts that both healthcare professionals and patients have in relation to aging and HIV. Dissipating those uncertainties around the link between aging and HIV might help to make aging with HIV less of an unknown and more like the next step in the journey of gay males living with HIV successfully.

This article sheds light on how discrimination is perceived while aging with HIV. In the literature the concept of “double-jeopardy”^41^ indicates discrimination in people with disabilities who grow old with a disability and Emlet^32^ has explored this concept in older gay males living with HIV in his qualitative analysis. However, from our analysis, it is possible to highlight how the jeopardy is not just doubled but it is intersectional, in line with Berger’s theoretical framework^42^ of intersectional stigma founded on the experiences of black women living with HIV, and based on the different identities of people. This intersectional stigma can also be experienced in different contexts such as healthcare, gay communities, online, etc Each of these contexts might be linked with needs of older gay males during their journey aging with HIV, needs that might not be met because of stigma perceived within those settings. The current data highlights the importance of exploring the sources of stigma and discrimination that are perceived in the local communities and institutional environments to develop awareness and tailored interventions to tackle stigma in more effective ways.

This study has several limitations that must be acknowledged when considering the findings presented above. Participants interviewed were mostly white and living in urban areas, thus the results are not transferable to people from other ethnic backgrounds who might hold different views. Another important point to consider is that this study has been conducted during the COVID-19 lockdown and this might have affected the answers given by participants.

Further research should be undertaken, including a more diverse sample in order to present different perspectives and experiences considering rural areas and people living with HIV from different ethnical and cultural background. Also, considering that this study was aimed at older gay males living with HIV, it would be interesting to explore the narratives emerging from other groups such as women and heterosexual men.

## Supplemental Material

sj-docx-1-jia-10.1177_23259582221144448 - Supplemental material for “It’s Still in the Test Tube and Finding out How the Experiment Ends… ”. A Qualitative Study on Health and Aging in Older Gay Males Living with HIV in England Click here for additional data file. Supplemental material, sj-docx-1-jia-10.1177_23259582221144448 for “It’s Still in the Test Tube and Finding out How the Experiment Ends… ”. A Qualitative Study on Health and Aging in Older Gay Males Living with HIV in England by Stefano Licchelli, Andrew King and Kimberley J. Smith in Journal of the International Association of Providers of AIDS Care (JIAPAC)

sj-docx-2-jia-10.1177_23259582221144448 - Supplemental material for “It’s Still in the Test Tube and Finding out How the Experiment Ends… ”. A Qualitative Study on Health and Aging in Older Gay Males Living with HIV in England Click here for additional data file. Supplemental material, sj-docx-2-jia-10.1177_23259582221144448 for “It’s Still in the Test Tube and Finding out How the Experiment Ends… ”. A Qualitative Study on Health and Aging in Older Gay Males Living with HIV in England by Stefano Licchelli, Andrew King and Kimberley J. Smith in Journal of the International Association of Providers of AIDS Care (JIAPAC)
