# Supplementary material for: “It’s Still in the Test Tube and Finding out How the Experiment Ends… ”. A Qualitative Study on Health and Aging in Older Gay Males Living with HIV in England
Source: J Int Assoc Provid AIDS Care. 2023 Jan 3;22:23259582221144448. doi: 10.1177/23259582221144448 (PMC9830087; doi:10.1177/23259582221144448)
Supplement: sj-docx-1-jia-10.1177_23259582221144448 - Supplemental material for “It’s Still in the Test Tube and Finding out How the Experiment Ends… ”. A Qualitative Study on Health and Aging in Older Gay Males Living with HIV in England [file sj-docx-1-jia-10.1177_23259582221144448.docx]

**Supplement Material - Interview schedule**

**Introduction**

Can you tell me more about yourself? (question)

*Your hobbies? (prompts, to be used if needed)*

*What do you like to do in you spare time?*

Have you always been living in this County?

Have you lived in different Counties during your life?

**Definition of health**

What does the word “*health*” means to you?

*How would you define health?*

*When you talk about health, what does it evoke in your mind?*

What are the things that make you say that you have a good or bad health?

*Do you think there are points that make you rate your health in a certain way?*

*Do you consider specific aspects when you rate your health? If yes, which aspects do you consider?*

Do you think your idea of health has changed over time? If yes, how?

*Can you identify when your idea of health changed? Was it in a particular moment of your life?*

*Are the different aspects that you consider to be a part of health changed?*

Please, describe how your health impact on your everyday life?

*Do you think living with HIV had had an impact on your daily activities?*

*Does your health status stop you in carrying out particular activities?*

**Ageing with HIV**

Can you think of particular challenges faced by people ageing with HIV?

*Difficulties that people might encounter.*

Can you think of particular needs that ageing with HIV might bring?

*Do you think there is a difference in ageing with or without HIV?*

*Housing, social, health needs?*

How do you find managing conditions other than HIV affects your health, if you have any?

*What other conditions do you have?*

*Is it easy to manage other conditions than HIV?*

*Do you disclose your status to other healthcare professionals?*

**Different kinds of discrimination in OPLWHIV**

Can you tell me of a time when you felt discriminated because of your age?

*What happened? When? What occasion?*

Can you tell me of a time when you felt discriminated because of HIV?

*What happened? When? What occasion?*

Do you think that ageing with HIV can lead to a new form of discriminations?

*Older people living with HIV would be more subjected to stigma and discrimination?*

*Do you think there is enough attention about stigma specifically on OPLWHIV?*

**Temporal perception of living with HIV**

Do you think the general perception of HIV is different compared with the past?

*Do you think there is more attention now?*

*Do people engage more or less in sexual health?*

*Do you think behaviours have changed?*

Do you think your perception of HIV has changed during your life?

*Are you looking at HIV differently now compared with the past?*

**Social and Community determinants**

How would you describe your experience of living with HIV in your local area?

*Do you find it difficult?*

*How do you think the people in your local area perceive HIV?*

*Does HIV stop you in accessing services in your local area?*

Can you tell me about services for PLWHIV in your local area? (e.g. charities, informal groups, peer-support, informative services, etc.)

*Have you used these kind of services?*

*Have you found them useful?*

*If there are no such services around you, what services would you like to have in your local community? Have you used other services such as online and distance services?*

What services do you think would be most useful in your local area to improve your health?

*Considering your idea of health, what would help you in improving your wellbeing?*

Considering your local area, which aspects should be tackled to improve your health?

*Which aspects, in your opinion, would improve your health if changed?*

*E.g. modifications to public transport, access to healthcare, more inclusive social policies, education, working conditions, etc.*

Do you think that living with HIV in your local area is different compared with a more urban/rural area?

*Do you think that there would be more/less occasion to socialise?*

*Do you think that other people would be more/less open to help people living with HIV?*

*Do you think there is more/less attention form the healthcare system?*

**Conclusion**

That is the end of our interview. Is there anything else that you would like to add?
