# Supplementary material for: “It’s Still in the Test Tube and Finding out How the Experiment Ends… ”. A Qualitative Study on Health and Aging in Older Gay Males Living with HIV in England
Source: J Int Assoc Provid AIDS Care. 2023 Jan 3;22:23259582221144448. doi: 10.1177/23259582221144448 (PMC9830087; doi:10.1177/23259582221144448)
Supplement: sj-docx-2-jia-10.1177_23259582221144448 - Supplemental material for “It’s Still in the Test Tube and Finding out How the Experiment Ends… ”. A Qualitative Study on Health and Aging in Older Gay Males Living with HIV in England [file sj-docx-2-jia-10.1177_23259582221144448.docx]

**Title and description for supplemental material item**

| **Title** | **Description** |
| --- | --- |
| Interview Schedule | Interview schedule used to investigate the concept of health in older gay males ageing with HIV as well as their experiences and perceptions of ageing with HIV. Supplemental Material for ““It’s still in the test tube and finding out how the experiment ends… ”. A qualitative study on health and ageing in older gay males living with HIV in England.” by [authors details] in Journal of the International Association of Providers of AIDS Care. |
